# Manganese Ferrite–Hydroxyapatite Nanocomposite Synthesis: Biogenic Waste Remodeling for Water Decontamination

**DOI:** 10.3390/nano12101631

**Published:** 2022-05-11

**Authors:** Jari S. Algethami, M. Shamshi Hassan, Ali Q. Alorabi, Nabil A. Alhemiary, Ahmed M. Fallatah, Yaser Alnaam, Saleh Almusabi, Touseef Amna

**Affiliations:** 1 Department of Chemistry, College of Science and Arts at Sharurah, Najran University, P.O. Box 1988, Najran 11001, Saudi Arabia; nafarhan@nu.edu.sa; 2 Department of Chemistry, College of Science, Albaha University, P.O. Box 1988, Albaha 65799, Saudi Arabia; aalorabi@bu.edu.sa; 3 Department of Chemistry, College of Science, Ibb University, Ibb P.O. Box 70270, Yemen; 4 Department of Chemistry, College of Science, Taif University, P.O. Box 11099, Taif 21944, Saudi Arabia; a.fallatah@tu.edu.sa; 5 Clinical Laboratory Sciences Department, Prince Sultan Military College of Health Sciences, KFMMC, P.O. Box 11099, Dhahran 31932, Saudi Arabia; yaser@psmchs.edu.sa (Y.A.); salmusabi@psmchs.edu.sa (S.A.); 6 Department of Biology, College of Science, Albaha University, P.O. Box 1988, Albaha 65799, Saudi Arabia

**Keywords:** water pollution, manganese ferrite/hydroxyapatite composite, bacteriostatic, pathogens, photocatalysis

## Abstract

Environmental pollution, especially water pollution caused by dyes, heavy metal ions and biological pathogens, is a root cause of various lethal diseases in human-beings and animals. Water purification materials and treatment methods are overpriced. Consequently, there is an imperative outlook observance for cheap materials for the purification of wastewaters. In order to fill up the projected demand for clean water, the present study aimed to make use of cost-effective and environmentally friendly methods to convert bone-waste from animals such as cows into novel composites for the decontamination of water. The bone-waste of slaughtered cows from the Najran region of Saudi Arabia was collected and used for the synthesis of hydroxyapatite based on the thermal method. The synthesized hydroxyapatite (Ca_10_(PO_4_)_6_(OH)_2_) was utilized to prepare a manganese ferrite/hydroxyapatite composite. The nanocomposite was categorized by diverse sophisticated procedures, for instance XRD, FE-SEM, EDX, TEM, UV, PL and FT-IR. This composite possesses outstanding photocatalytic activity against methylene blue dye, which is a common pollutant from industrial wastes. Moreover, the synthesised composite revealed exceptional bacteriostatic commotion towards *E. coli* and *S. aureus* bacteria, which are accountable for acute waterborne infections. The outcome of this study demonstrated that the integration of manganese ferrite into hydroxyapatite significantly intensified both antimicrobial and photocatalytic actions when compared to the virgin hydroxyapatite.

## 1. Introduction

Currently, hygienic water is a protuberant global problem. Safe water is a prerequisite for maintaining livelihood as well as an ecological niche. Nevertheless, negligence and misgovernance of water reservoirs have drastically threatened the accessibility of fresh water. Millions of people die annually due to contaminated water as well as water-borne infections [1]. Undeniably, microorganisms account for water-associated illnesses. The occurrence of coliforms is a marker of current defecation effluence. The outbreaks of water-related microbial infections are prime sources of death [2]. On the other hand, inorganic materials and heavy metals, although not very detrimental in lesser quantities, operate as noxious waste with time in water. Similarly, organic materials enter water bodies through leaching, desecrate dumping, human activities or industrialized catastrophes [3]. This type of contaminated water, particularly in elevated absorption, might result in acute health issues and even the death of human beings and other life forms. Additionally, organic contaminants and poisonous substances from industrial wastes result in an acute logjam of environmental and health concerns. Even a scanty amount of dyes may adversely affect aquatic fauna and flora as they are carcinogenic in nature. In particular, cationic dyes are found to be more toxic than anionic dyes owing to their synthetic nature and aromatic ring configuration with delocalized electrons [4]. Moreover, the occurrence of these dyes worsens the productivity of agricultural land [5]. Henceforth, the removal of dye molecules from polluted water is of crucial prominence prior to its release into water bodies.

Considering projected demand for clean water worldwide and specifically in Saudi Arabia, there is an important urge for the development of cheap and efficient methods as well as materials for the purification of wastewater. Thus, the present study envisages the development of cost-effective material (hydroxyapatite) from animal bones. Previously, sheep-bone charcoal and activated carbons have been investigated for the adsorption of mercury ions [3]. Moreover, animal-fillet charcoal has been applied as an adsorbent for the elimination of mercury from polluted waters. Interestingly, magnetic nanocomposite from waste animal-bone biochar has also been utilized for the exclusion of divalent metals from wastewater [6]. Recently, various organic/inorganic hydroxyapatite composites have been employed to take away diverse coloring effluents. For instance, Panneerselvam et al. used iron- and cobalt-based hydroxyapatite as well as pristine hydroxyapatite (HAP) for the removal of Congo red dye [7]. Manatunga et al. utilized HAP composites as well as pristine HAP to adsorb Acid yellow, which indicates that the ligand attached to the HAP had an influence on its adsorptive capacity [8]. Hou et al. developed a HAP-Chitosan composite for Congo red removal. Guan et al. coated HAP with polyalcohol to eliminate Congo red, methyl blue and orange [9]. Increased photocatalytic removal of NO by the TiO_2_-HAP composite was reported by Yao et al. [10]. Additionally, other studies on the photocatalytic degradation of dyes by nanotextured HAP have also been reported [11,12]. Consequently, the literature suggests that HAP is an important material for the removal of toxic materials from wastewater. In view of an increased aspect ratio, insignificant solubility in water, abundant hydroxyl assemblies, accessibility, economics, and an eco-friendly and facile preparation process, HAP has widely been exploited as a sorbent for the elimination of noxious components from contaminated waters [13]. Additionally, HAP develops bonds with diverse sizes of organic molecules. Customarily, HAP is delivered in powder form or pellets, which confines its industrial uses. Therefore, HAP-containing composites are extensively being explored. It is strongly believed that HAP-based composite materials will be a suitable alternative [14] to the expensive materials commonly used to clean wastewater. Furthermore, some noble procedures have been attempted to scale-up HAP production, for instance Fluidinova in collaboration with the Instituto de Engenharia Biomedica has established and patented a scale-up process for the fabrication of high-quality materials comprising HAP nanoparticles [15]. Likewise, an in situ crystallization procedure consuming a facile reaction of Ca(OH)_2_ and H_3_PO_4_ has been exploited for the generation of large-scale HAP nanoparticles [16].

On the other hand, MnFe_2_O_4_ is considered one of the finest magnetic-material substitutes for Fe_3_O_4_ owing to its outstanding physicochemical assets [17,18]. Nevertheless, bulk MnFe_2_O_4_ could not efficiently eradicate heavy metal ions and dyes. In this regard, nano-sized magnetic particles overcome these glitches and can yield bigger specific surface areas, which produce superior adsorption or photocatalytic capacities for the elimination of contamination [19,20]. Nanotextured ferrite is a type of composite oxide with the key constituents of trivalent iron oxides [21]. These benevolent materials have triggered eclectic public concern due to superior physical and chemical features, viz. unwavering chemical properties, reasonable saturated magnetic field and so on. 

Instigated by the remarkable properties of MnFe_2_O_4_ and biogenic HAP, in the current study, we recycled waste-bones to design an economical MnFe_2_O_4_ assimilated HAP nanocomposite for the decontamination of water from hazardous biological and chemical pollutants.

Wastewater treatment methods are very expensive and challenging. Nowadays, photocatalytic degradation of dyes is an emerging technique accomplishing colossal consideration in handling wastewaters. It is an innovative procedure established while bearing in mind the failure of various earlier water-management methods. Nevertheless, many photocatalysts can only be activated by ultraviolet light, but UV light is just around 5% of the solar spectrum whereas visible light is ~45%. Accordingly, for effectual solar-energy exploitation, it is important to shift the light response from UV to visible light. Additionally, intense UV-light repeatedly generates ample amounts of reactive by-products. This challenge can also be overcome by means of visible light as an energy resource. The mechanistic procedure of visible-light response to visible-light-active photocatalysts is almost identical to that of UV-light-active photocatalysts. The only variance is less photon energy requirement to activate the photocatalytic cycle that perhaps results in superior selectivity [22]. It has also been recognized that the material used should be both non-toxic and unaffected by photooxidative disintegration [5,12]. Herein, HAP-MnFe_2_O_4_ nanocomposites were efficaciously synthesized and characterized in detail. These nanocomposites were used for the degradation of methylene blue (MB) dye by the visible-light photocatalysis method.

## 2. Materials and Methods

### 2.1. Synthesis of Hydroxyapatite (HAP)

The thrown-away carcasses were collected from a slaughterhouse in Najran, Saudi Arabia. These carcasses were splashed comprehensively with water and prudently scrubbed with acetone to eliminate fat, connective tissue and extra contaminations. Afterwards, the sanitized bones were dehydrated at high temperature (160 °C) for 48 h. Additionally, the desiccated carcasses were calcined in a furnace at elevated temperature (600 °C) for the extraction of biogenic HAP [14].

### 2.2. Synthesis of HAP-MnFe_2_O_4_ Nanocomposite

Briefly, approximately 2 mmol of FeCl_3_·6H_2_O and 1 mmol of MnCl_2_·4H_2_O were dispersed into 70 mL distilled water (DW) to process a homogenous metal–ion solution while stirring. During stirring, sodium hydroxide (2 M) liquefied with 10 mL DW was supplemented into the above metal–ion mixture drop-by-drop until pH~12. The attained intermediate was strained and splashed with distilled water until the pH of the filtrate turned neutral. Afterwards, an equivalent quantity of HAP was disseminated into the solution (1:1 mass ratio) with continuous stirring to produce a consistent dispersion at room temperature (RT). The assimilated solution was placed into a Teflon-lined stainless autoclave. The autoclave was then retained into an oven and placed at 200 °C for 12 h and was allowed to cool at RT. The ultimate product was centrifuged and splashed thrice by DW and absolute ethanol. Finally, the products were eventually dehydrated in an oven at 60 °C for 12 h.

### 2.3. Characterization

The crystalline phases of HAP and HAP-MnFe_2_O_4_ nanocomposite were scanned by X-ray diffraction (XRD, Rigaku D/Max-2550, λ = 0.154 18 nm). The outward arrangement and dimensions have been described using Field emission scanning electron microscopy (FESEM) from SHIMADZU Japan (SSX-550) furnished with Energy-dispersive X-ray (EDX). Infrared data were revealed on a VERTEX 70 Fourier transform infrared (FTIR) spectrometer (Bruker, Ettlingen, Germany) at a resolution of 4 cm^−1^ and a scan rate of 0.75 Hz using ATR mode. Ultra-violet−visible diffuse-reflectance spectra (UV−vis DRS) were acquired on an UV−vis spectrophotometer (UV-2550, Shimadzu, Kyoto, Japan). Photoluminescence (PL) data were obtained at room temperature on an F-7000 fluorescence spectrophotometer (Hitachi, Tokyo, Japan) with an excitation wavelength of 325 nm. The microscopic features and crystalline pattern of the composite were examined by Transmission electron microscopy (TEM) (H-7650, Hitachi, Japan)

### 2.4. Assessment of Photocatalytic Activity

The photocatalytic performance of pure HAP and HAP/MnFe_2_O_4_ nanocomposite photocatalysts was estimated through disintegration of the MB aqueous solution in visible light (λ ≥ 400 nm) with a 450 W mercury lamp. Fifty milligrams of the sample were supplemented in 200 mL of the MB aqueous solution (20 ppm). Before irradiation of the light, the solution was magnetically stirred for 30 min in the dark to reach adsorption−desorption equilibrium. After irradiation for a particular time, 3 mL of solution was taken out and centrifuged. The filtrate was examined by recording variations of maximum absorption band (668 nm) using UV-vis spectrophotometer (Thermo Fisher Scientific, Weltham, MA, USA).

### 2.5. Antimicrobial Potential of HAP and HAP/MnFe_2_O_4_ Nanocomposite

The standard bacteria from American Type Culture Collection (ATCC) (*E. coli* 25922 and *S. aureus* 25923) were chosen from stored (at −80 °C) strains. In order to make usage of lyophilized bacteria, microorganisms were revived on an agar medium in order to evaluate the viability and pureness of the selected strains. Furthermore, revived axenic microorganisms were retained on a nutrient agar for use in a bacterial susceptibility test. The antimicrobial action of virgin HAP and HAP-MnFe_2_O_4_ nanocomposites was screened against abovementioned strains. The bacterial stock cultures were retained on Muller–Hinton agar (MHA) plates. The loopful of absolutely grown culture from plates was cultivated into normal saline (5 mL, 85% NaCl), and the count was 1 × 10^6^ CFU/mL. To establish the antimicrobial potential, pristine HAP and HAP-MnFe_2_O_4_ nanocomposite were diluted serially to obtain four varied concentrations and were then screened. The experimental concentrations applied herein were 0, 50, 100 and 200 µg/mL. The kinetics were studied at 37 °C with an rpm of 150, maintaining a regular time interval (4 h) based on an inspection of OD using a spectrophotometer. The alteration in absorbance was premeditated at 600 nm by a UV-spectrophotometer. The aforementioned pathogenic strains were grown with specific quantity (two-fold dilution) of the selected nanocomposites in order to verify the minimum inhibitory concentration (MIC).

## 3. Results

**Figure 1:**
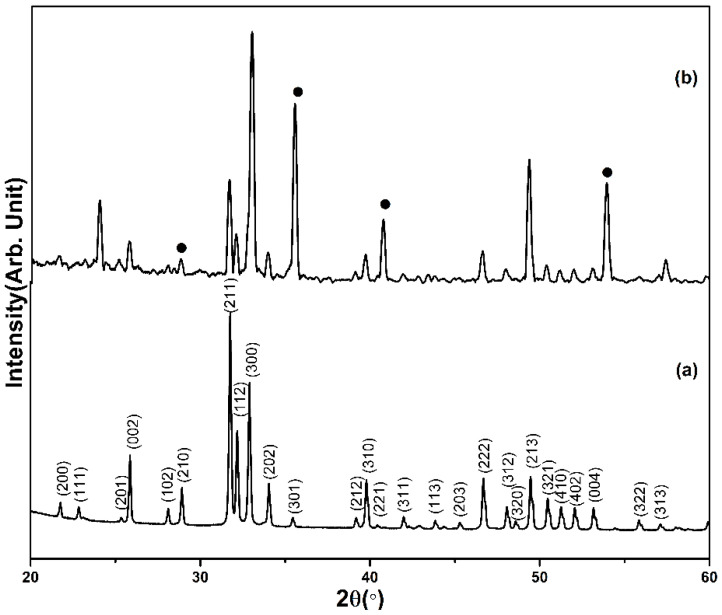
XRD pattern of (**a**) HAP and (**b**) HAP-MnFe_2_O_4_ nanocomposite.

The crystalline configuration of synthesized pure and nanocomposites was categorized by XRD. The XRD spectra of pure HAP and HAP-MnFe_2_O_4_ composite are shown in Figure 1. For the pure HAP, all diffraction peaks confirm the formation of polycrystalline HAP (JCPDS no. 09-0432) [23]. No impurity peaks, calcium hydroxide or phosphate were observed in the HAP spectrum (Figure 1a). For the composite sample, the diffraction peaks of MnFe_2_O_4_ were observed along with HAP peaks. The diffraction peak can be indexed to the cubic structure of manganese ferrite (JCPDS no. 88-1965) (Figure 1b) [24]. The results suggest the coexistence of HAP and MnFe_2_O_4_; no impurities are found in the nanocomposite spectrum.

**Figure 2:**
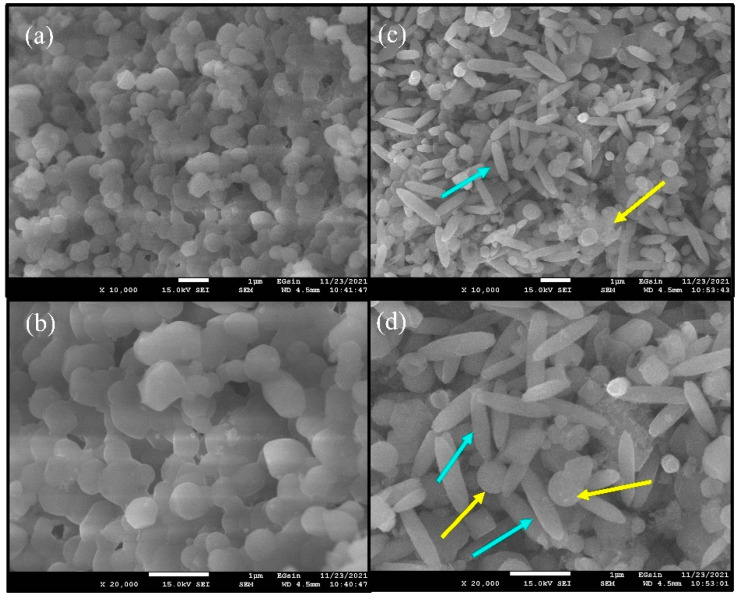
FE-SEM images of (**a**,**b**) HAP and (**c**,**d**) HAP-MnFe_2_O_4_ at low and high magnifications. The yellow arrows indicate HAP nanoparticles, whereas the cyan arrows MnFe_2_O_4_ in the HAP-MnFe_2_O_4_ nanocomposite.

Figure 2 shows FESEM micrographs of HAP and the HAP-MnFe_2_O_4_ composite with different magnifications. From Figure 2a,b, it can be perceived that HAP nanoparticles are round shaped, having a typical diameter of around 500 nm, whereas the composite sample shows emblematic a rice-shape nanostructure along with the round nanoparticles. The rice-shape structure is probably MnFe_2_O_4_, having a diameter of about 200 nm (Figure 2c,d).

**Figure 3:**
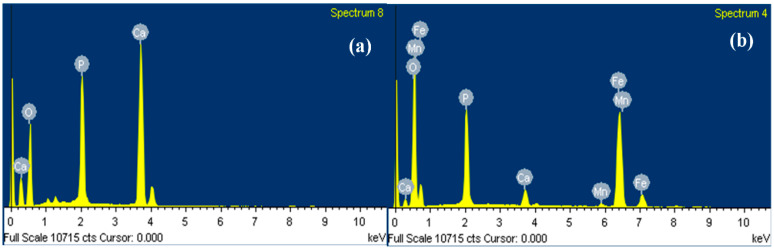
EDX spectra of (**a**) HAP and (**b**) HAP-MnFe_2_O_4_ nanocomposite.

The chemical configuration of the samples was further identified by EDX analysis (Figure 3). The EDX result shown in Figure 3a clearly identifies the peaks of Ca, P and O, which confirms the presence of hydroxyapatite, while the composite sample shows the presence of Mn and Fe in addition to Ca, P and O (Figure 3b).

**Figure 4:**
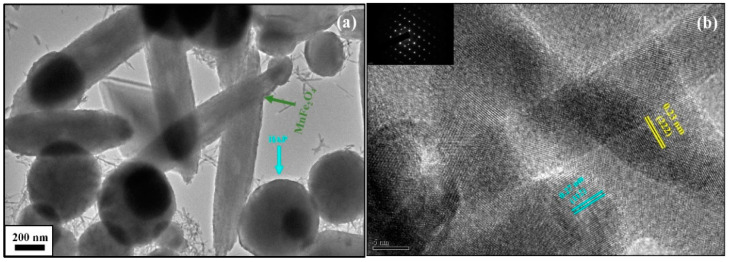
(**a**) TEM and (**b**) HR-TEM spectra of HAP-MnFe_2_O_4_ nanocomposite; *inset* shows SAED pattern.

Figure 4 demonstrated the TEM and high-resolution TEM images of HAP-MnFe_2_O_4_. It can be seen from the micrographs that the composite has spherical HAP nanoparticles with MnFe_2_O_4_ rice-shaped nanostructures (Figure 4a). The HAP particles have diameters of around 500 nm and ~200 nm for MnFe_2_O_4_, which are consistent with the SEM results. Figure 4b revealed an HR-TEM image having d-spacings of 0.23 and 0.27 nm, which corresponded to the (222) and (112) plane of MnFe_2_O_4_ and HAP, respectively, thus confirming the formation of a heterostructure. The selected area electron diffraction (SAED) pattern (*inset* Figure 4b) showed the multicrystalline phase of the HAP-MnFe_2_O_4_ composite.

**Figure 5:**
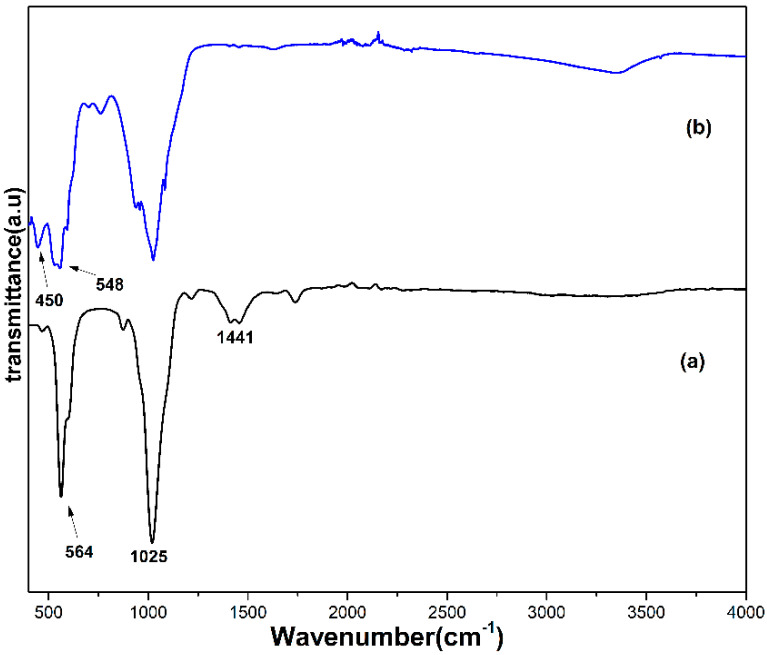
FT−IR spectra of (**a**) HAP and (**b**) HAP−MnFe_2_O_4_.

Figure 5 demonstrates the FTIR spectra of virgin HAP and MnFe_2_O_4_-HAP composite. For unalloyed HAP, the main peaks at 564 show the characteristic PO_4_^3−^ vibrations due to O–P–O winding mode, and the crowning at 1025 cm^−1^ could be allocated to anti-symmetric stretching vibrations of the phosphate group [25,26]. Furthermore, the weak peaks at about 1441 cm^−1^ were allotted to absorption bands of CO_3_^2−^, representing carbonate ions’ formation due to reaction atmosphere (Figure 5a). The broad peak at 3000−3500 cm^−1^ in both spectra endorsed to the stretching vibration of O−H of substantially adsorbed water [27,28]. Figure 4b shows the absorption ensembles at 548 cm^−1^ assigned to the stretching vibration of manganese–oxygen (Mn-O) links, which were produced by MnFe_2_O_4_ in an octahedral shape [29] and a band at 450 cm^−1^ matching the vibration of metal–oxygen (Mn-O and Fe-O) bonds at octahedral loci from MnFe_2_O_4_ [30]. The results further confirmed the formation of MnFe_2_O_4_ along with HAP in the composite (Figure 5b).

**Figure 6:**
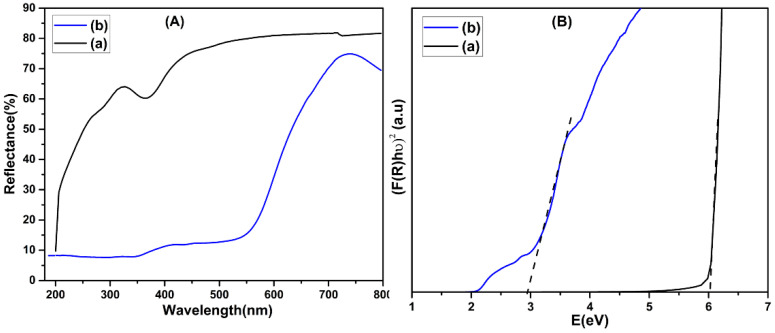
(**A**) UV–Vis diffuse reflection spectra and (**B**) plot of the transformed Kubelka–Munk function versus the gap energy of sample (**a**) HAP and (**b**) HAP−MnFe_2_O_4_ nanocomposite.

Figure 6 shows the UV-DRS spectra of pure HAP and HAP-MnFe_2_O_4_ composites. The reflectance edge of HAP is around 200 nm, which is in the ultraviolet range. After the addition of MnFe_2_O_4_, the reflectance edge of the composites shifts to a higher wavelength in the visible region (Figure 6A). The band gaps of HAP and HAP-MnFe_2_O_4_ are found to be 2.95 eV and 6.02 eV, as shown in Figure 6B. The narrow band gap of the composites can significantly utilize more visible light, which can be advantageous for improving the photocatalytic activity.

**Figure 7:**
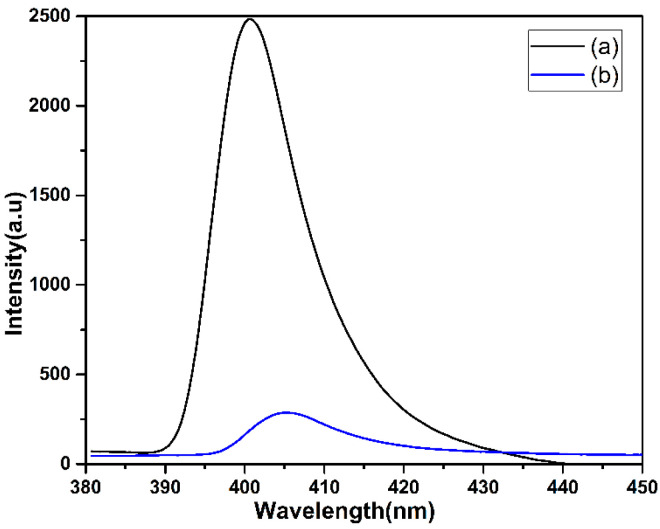
PL spectra of (**a**) HAP and (**b**) HAP−MnFe_2_O_4_ nanocomposite.

Photoluminescence (PL) spectra of synthesized pure HAP and HAP−MnFe_2_O_4_ composite were applied to examine the separation of charge carriers (Figure 7). Both the samples exhibited an emission peak at around 400 nm. In comparison to HAP, the HAP-MnFe_2_O_4_ exhibits a substantial decrease in intensity of PL, which signifies an effective charge transfer inside the HAP-MnFe_2_O_4_ composite and a decrease in electron−hole pair recombination. In response, this will assist in the efficient transfer of electrons and holes at the surface of the heterostructure, eventuating an increase in photo-degradation efficiency.

**Figure 8:**
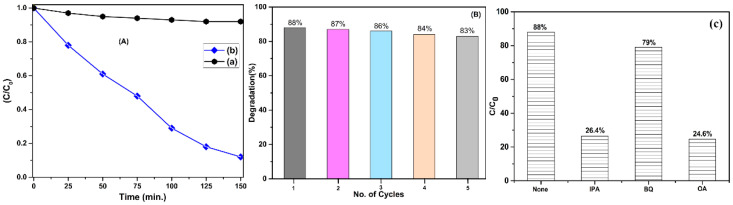
(**A**) Photocatalytic degradation of MB in the presence of sample (a) HAPs and (b) HAP-MnFe_2_O_4_. (**B**) Photodegradation efficiency of MB over HAP−MnFe_2_O_4_ in different recycles. (**C**) Trapping experiments of active species in the photocatalytic reaction.

The photodegradation efficiency of synthesized HAP and HAP−MnFe_2_O_4_ composites were assessed by MB dye degradation using visible light. The composite sample displayed much better photodegradation efficiency compared to pristine HAP (Figure 8A). HAP-MnFe_2_O_4_ composites showed about 88% dye removal after 150 min of light exposure. Earlier studies have also reported the photocatalytic degradation of industrial dyes such as crystal violet (77%) and Congo red (87%) by HAP obtained from recycled fish bones [12]. In an investigation, a nickel/hydroxyapatite/cobalt ferrite composite was used as a heterogeneous catalyst for the degradation of MB and methyl orange. Interestingly, the catalyst displayed a degradation of ~90% (MO) and 99.1% (MB) in the existence of hydrogen peroxide [31]. Furthermore, in another study, HAP spheres were analyzed for the degradation of MB under UV irradiation and ~75% degradation efficacy on MB was observed [4].

The high efficiency of the composite sample can be ascribed to the formation of the heterostructure between HAP and MnFe_2_O_4_. The recyclability of composite was also investigated for MB degradation (Figure 8B). After reusing it for five cycles, the composite showed a slight decrease in the photodegradation efficiency of MB, and it remained around 83%, which suggests excellent stability of the composite material.

To understand the reaction mechanism, trapping experiments were performed in a similar procedure except that different radical scavengers (1 mmol) were added into the photocatalytic reaction solution under visible light irradiation for 150 min, as shown in Figure 8C. To identify major reactive species formed in the present system, trapping experiments were performed with ammonium oxalate (AO), isopropanol (IPA) and benzoquinone (BQ) as scavengers to quench h^+^, •OH and •$O_{2}^{-}$, respectively. As shown in the figure, the removal rate of MB was 26.4% and 24.6% in the presence of IPA and OA, respectively, whereas the removal rate was only 79% in the presence of BQ. Therefore, it was found that h^+^ and •OH are the main active species for the HAP-MnFe_2_O_4_ nanocomposite. Electron and hole pairs are produced when visible light is irradiated on MnFe_2_O_4_; the generated electrons are then transferred to the surface of HAP nanoparticles. It reacts with adsorbed oxygen molecules to produce active oxygen species $O_{2}^{-}$•; it again combines with H^+^ ions to produce HO_2_^•^, which ultimately combines with trapped electrons to produce OH^•^ [32]. An excess of holes remains in MnFe_2_O_4_ surfaces, reacting with H_2_O or OH^−^ to produce active species such as OH^•^. These active radicals ($O_{2}^{-}$• and OH^•^) and holes can be utilized for the decomposition process of MB dye as follows:

hν (Visible) + MnFe_2_O_4_ → h^+^ + e^−^ (1)

(2) $$(\mathrm{HAP})\;e^{-}+O_{2}\;\to \;O_{2}^{-}•$$

h^+^ + H_2_O → H^+^ + OH^•^ (3)

(4) $$h^{+}+O_{2}^{-}•+{\mathrm{OH}}^{•}+\mathrm{MB}\;\mathrm{Dye}\;\to \;\mathrm{Degraded}\;\mathrm{product}\;$$

**Figure 9:**
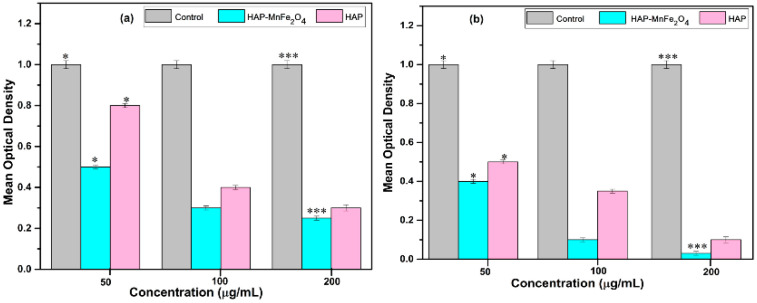
Bar graphics of (**a**) *E. coli* culture augmented with different quantities of HAP and HAP-MnFe_2_O_4_ nanocomposite (optimal expansion signifies *E. coli* and *S. aureus* in culture broth without HAP and HAP-MnFe_2_O_4_). Substantial difference (* *p* ≤ 0.05) was perceived in bacterial growth inhibition among control group and treatments at all concentrations. Significant difference in bacteriostatic impact with HAP and HAP-MnFe_2_O_4_ at higher amount was visualized. * *p* ≤ 0.05, *** *p* ≤ 0.001 significantly dissimilar from untreated control.

The virgin HAP and HAP-MnFe_2_O_4_ nanocomposites were also evaluated for their consumption of varying amounts of *S. aureus* and *E. coli*, applying our earlier established assay [33,34]. The synthesized nanocomposites demonstrated estimable antimicrobial activity against both Gram-negative *E. coli* and Gram-positive *S. aureus*, with MICs of 50 μg/mL. The outcomes are revealed in Figure 9. On the other hand, comparatively mild antibacterial action was observed with HAP at the identical concentration. Evidently, an insignificant bacteriostatic effect was observed at a low concentration with HAP against *E. coli*, but better performance was observed with nanocomposites against *S. aureus*. The enhanced activity of the HAP-MnFe_2_O_4_ nanocomposite was credited with morphological features with enlarged surfaces [35] as well as a synergism [36] between HAP and MnFe_2_O_4_. The hypothesis could be that, at first, HAP and HAP-MnFe_2_O_4_ nanocomposite probably interacted with the bacterial wall and membrane, later on dispersing to the interior of the cell, instigating leakage by distracting the cell’s contents.

**Scheme 1:**
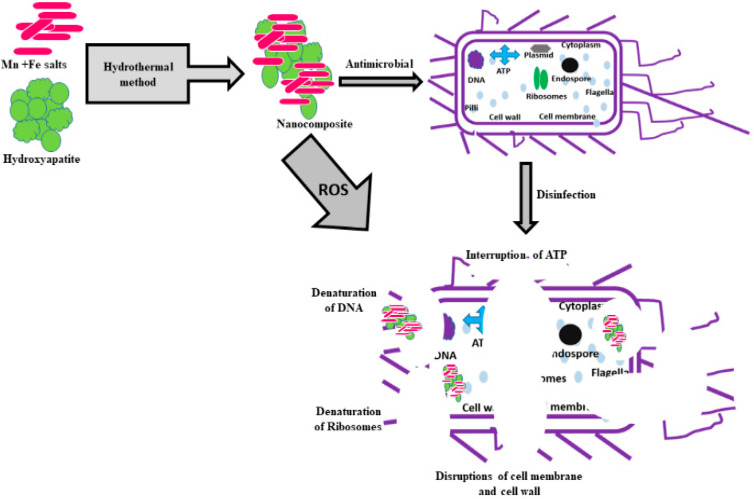
Plausible antibacterial mechanism of HAP-MnFe_2_O_4_ nanocomposites.

A number of earlier investigations have already reported that nanocomposites can attach to the outer membrane of bacteria through electrostatic contact and cause disturbance of membrane, suppress periplasmic enzymes, and rupture bacteria, eventually restricting protein synthesis [37]. Generally, the antimicrobial action of HAP is interrelated with the discharge of OH^−^ ions in an aqueous medium. Hydroxyl ions are extremely oxidant-free radicals that express high reactivity with a number of biomolecules. The reactivity is extraordinary and unselective; therefore, these free radicals hardly disperse from spots of creation. Convincingly, their deadly impact on bacterial cells is perhaps owed to these mechanisms, for instance impairment of the bacterial cytoplasmic membrane, protein disintegration and destruction to DNA [37], as aforementioned. Furthermore, the electron transfer taking place on a facet of HAP-MnFe_2_O_4_ can produce free oxidative radicals, which are lethal to bacteria [36] (Scheme 1).

The superior effectiveness of HAP-MnFe_2_O_4_ nanocomposites towards *S. aureus* is accredited to the dissimilarity in the cell structures of two strains [35,38]. The conformation and arrangement of the cell is the principally accountable factor for variances in their sensitivity. Conclusively, the aforementioned mechanisms of HAP nanoparticles in influence with MnFe_2_O_4_ nano rice displayed promising antibacterial results in this work.

## 4. Conclusions

In this paper, HAP and HAP-MnFe_2_O_4_ nanocomposite photocatalysts were primed on the basis of the hydrothermal approach. The characteristic results of XRD, FESEM, TEM and FTIR indicated that the composite was successfully fabricated. The photodegradation experiments of MB revealed high efficiency of the composite photocatalyst. In addition, the prepared photocatalysts displayed good recyclability and stability under visible light irradiation. The enriched photocatalytic and bactericidal activity of HAP-MnFe_2_O_4_ nanocomposites was predominantly credited to synergetic effects between HAP and MnFe_2_O_4_.

Conclusively, the outcomes of this study delivered herein not only suggest an extremely competent and unwavering photocatalytic material for the purification of wastewaters from chemical and biological pollutants but also shed light on the recycling of waste materials (unwanted bones) in Najran city in particular and worldwide in general.

Our study specifically offers the possibility for further investigations on comparable heterostructure materials by recycling wastes and for environmental remediation.

More to the point, the efficacy of photocatalysts can be enhanced by diverse approaches such as modification in synthesis methods as well as mutable morphologies and by doping of the various natural/organic compounds with suitable metals, metal oxides, etc.

In the present investigation, visible light photocatalysis was used for wastewater treatment. This incredible approach was also found useful for the destruction of pathogenic microorganisms such as bacteria. Therefore, this procedure may result in the mineralization of hazardous biological and chemical pollutants. The antibacterial and photocatalytic removal mechanism of MB over HAP-MnFe_2_O_4_ nanocomposites is exemplified in detail.

This study suggests as high as 88% visible light photocatalytic activity and thus that this material can be reserved as a promising water purifying material. However, before using this material (HAP-MnFe_2_O_4_ nanocomposites) commercially, the biocompatibility with aquatic fauna and flora as well as with human cells should be tested.

Nonetheless, the application of visible light photocatalysis has good potential for improving water quality and henceforth global water scarceness. However, to meet practical challenges for industrial applications, more studies need to be conducted on the photoreactor’s design, capacity, competence, reliability and ease of use.

## Data Availability

Not applicable.
